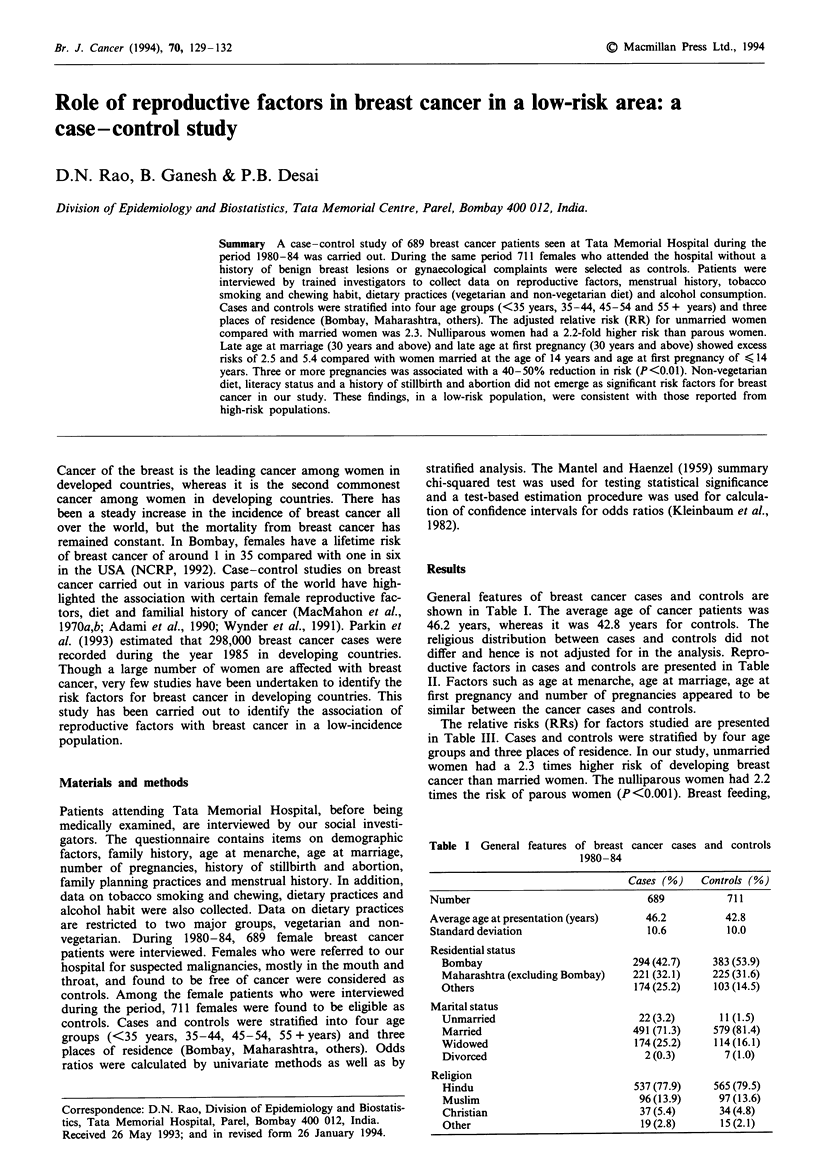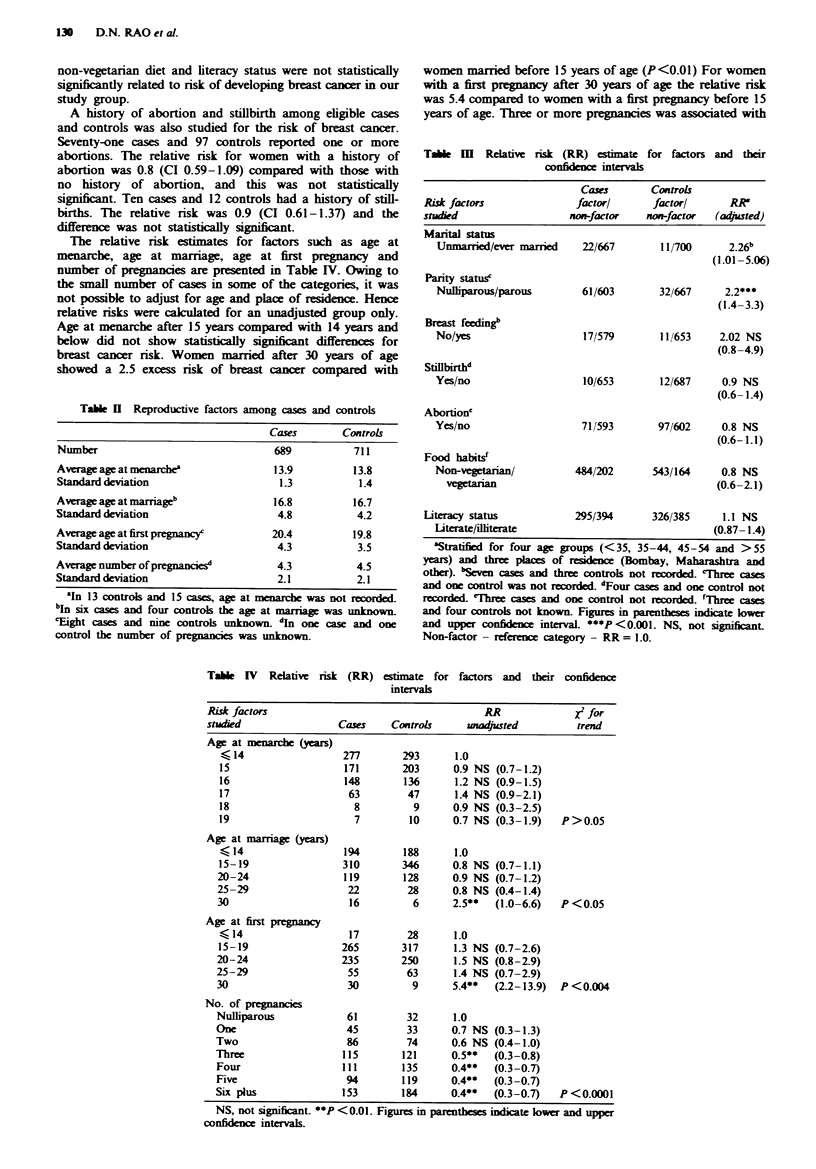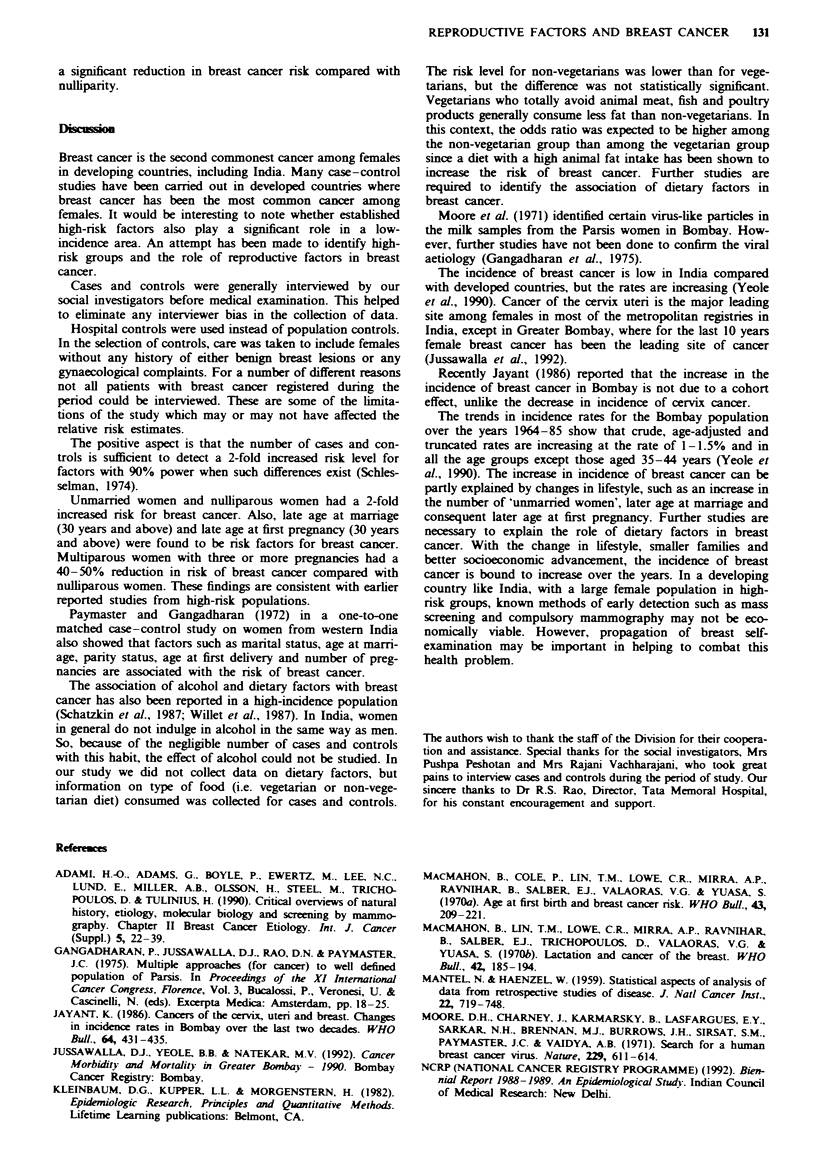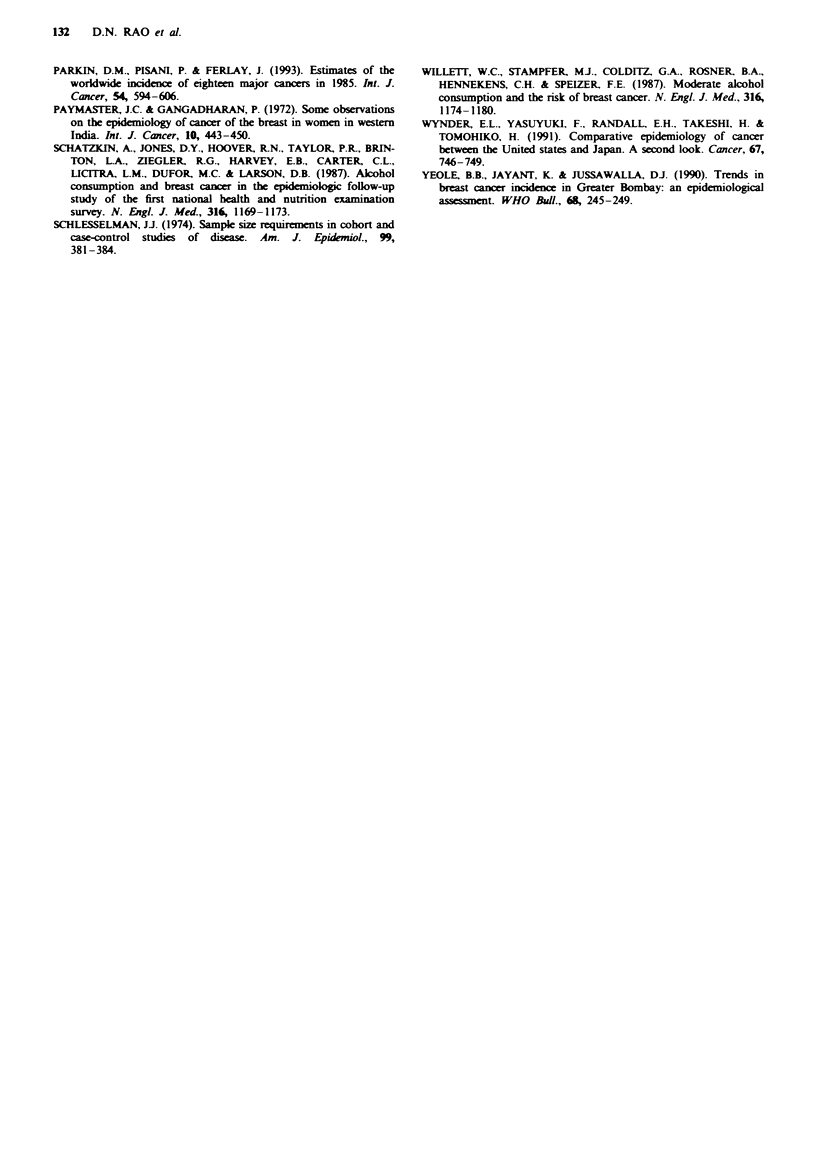# Role of reproductive factors in breast cancer in a low-risk area: a case-control study.

**DOI:** 10.1038/bjc.1994.261

**Published:** 1994-07

**Authors:** D. N. Rao, B. Ganesh, P. B. Desai

**Affiliations:** Division of Epidemiology and Biostatistics, Tata Memorial Centre, Parel, Bombay, India.

## Abstract

A case-control study of 689 breast cancer patients seen at Tata Memorial Hospital during the period 1980-84 was carried out. During the same period 711 females who attended the hospital without a history of benign breast lesions or gynaecological complaints were selected as controls. Patients were interviewed by trained investigators to collect data on reproductive factors, menstrual history, tobacco smoking and chewing habit, dietary practices (vegetarian and non-vegetarian diet) and alcohol consumption. Cases and controls were stratified into four age groups (< 35 years, 35-44, 45-54 and 55+ years) and three places of residence (Bombay, Maharashtra, others). The adjusted relative risk (RR) for unmarried women compared with married women was 2.3. Nulliparous women had a 2.2-fold higher risk than parous women. Late age at marriage (30 years and above) and late age at first pregnancy (30 years and above) showed excess risks of 2.5 and 5.4 compared with women married at the age of 14 years and age at first pregnancy of < or = 14 years. Three or more pregnancies was associated with a 40-50% reduction in risk (P < 0.01). Non-vegetarian diet, literacy status and a history of stillbirth and abortion did not emerge as significant risk factors for breast cancer in our study. These findings, in a low-risk population, were consistent with those reported from high-risk populations.


Br. J. Cancer (1994), 70, 129-132 © Macmillan Press Ltd., 1994

### Role of reproductive factors in breast cancer in a low-risk area: a case-control study

D.N. Rao, B. Ganesh & P.B. Desai

*Division of Epidemiology and Biostatistics, Tata Memorial Centre, Parel, Bombay 400 012, India.*

**Summary** A case-control study of 689 breast cancer patients seen at Tata Memorial Hospital during the period 1980-84 was carried out. During the same period 711 females who attended the hospital without a history of benign breast lesions or gynaecological complaints were selected as controls. Patients were interviewed by trained investigators to collect data on reproductive factors, menstrual history, tobacco smoking and chewing habit, dietary practices (vegetarian and non-vegetarian diet) and alcohol consumption. Cases and controls were stratified into four age groups (<35 years, 35-44, 45-54 and 55 + years) and three places of residence (Bombay, Maharashtra, others). The adjusted relative risk (RR) for unmarried women compared with married women was 2.3. Nulliparous women had a 2.2-fold higher risk than parous women. Late age at marriage (30 years and above) and late age at first pregnancy (30 years and above) showed excess risks of 2.5 and 5.4 compared with women married at the age of 14 years and age at first pregnancy of ≤14 years. Three or more pregnancies was associated with a 40-50% reduction in risk ($P<0.01$). Non-vegetarian diet, literacy status and a history of stillbirth and abortion did not emerge as significant risk factors for breast cancer in our study. These findings, in a low-risk population, were consistent with those reported from high-risk populations.

Cancer of the breast is the leading cancer among women in developed countries, whereas it is the second commonest cancer among women in developing countries. There has been a steady increase in the incidence of breast cancer all over the world, but the mortality from breast cancer has remained constant. In Bombay, females have a lifetime risk of breast cancer of around 1 in 35 compared with one in six in the USA (NCRP, 1992). Case-control studies on breast cancer carried out in various parts of the world have highlighted the association with certain female reproductive factors, diet and familial history of cancer (MacMahon *et al.*, 1970*a,b*; Adami *et al.*, 1990; Wynder *et al.*, 1991). Parkin *et al.* (1993) estimated that 298,000 breast cancer cases were recorded during the year 1985 in developing countries. Though a large number of women are affected with breast cancer, very few studies have been undertaken to identify the risk factors for breast cancer in developing countries. This study has been carried out to identify the association of reproductive factors with breast cancer in a low-incidence population.

### Materials and methods

Patients attending Tata Memorial Hospital, before being medically examined, are interviewed by our social investigators. The questionnaire contains items on demographic factors, family history, age at menarche, age at marriage, number of pregnancies, history of stillbirth and abortion, family planning practices and menstrual history. In addition, data on tobacco smoking and chewing, dietary practices and alcohol habit were also collected. Data on dietary practices are restricted to two major groups, vegetarian and non-vegetarian. During 1980-84, 689 female breast cancer patients were interviewed. Females who were referred to our hospital for suspected malignancies, mostly in the mouth and throat, and found to be free of cancer were considered as controls. Among the female patients who were interviewed during the period, 711 females were found to be eligible as controls. Cases and controls were stratified into four age groups (<35 years, 35-44, 45-54, 55 + years) and three places of residence (Bombay, Maharashtra, others). Odds ratios were calculated by univariate methods as well as by stratified analysis. The Mantel and Haenzel (1959) summary chi-squared test was used for testing statistical significance and a test-based estimation procedure was used for calculation of confidence intervals for odds ratios (Kleinbaum *et al.*, 1982).

Correspondence: D.N. Rao, Division of Epidemiology and Biostatistics, Tata Memorial Hospital, Parel, Bombay 400 012, India.
Received 26 May 1993; and in revised form 26 January 1994.

### Results

General features of breast cancer cases and controls are shown in Table I. The average age of cancer patients was 46.2 years, whereas it was 42.8 years for controls. The religious distribution between cases and controls did not differ and hence is not adjusted for in the analysis. Reproductive factors in cases and controls are presented in Table II. Factors such as age at menarche, age at marriage, age at first pregnancy and number of pregnancies appeared to be similar between the cancer cases and controls.

The relative risks (RRs) for factors studied are presented in Table III. Cases and controls were stratified by four age groups and three places of residence. In our study, unmarried women had a 2.3 times higher risk of developing breast cancer than married women. The nulliparous women had 2.2 times the risk of parous women ($P<0.001$). Breast feeding,

**Table I** General features of breast cancer cases and controls 1980-84

| | *Cases (%)* | *Controls (%)* |
|---|---|---|
| Number | 689 | 711 |
| Average age at presentation (years) | 46.2 | 42.8 |
| Standard deviation | 10.6 | 10.0 |
| Residential status | | |
| Bombay | 294 (42.7) | 383 (53.9) |
| Maharashtra (excluding Bombay) | 221 (32.1) | 225 (31.6) |
| Others | 174 (25.2) | 103 (14.5) |
| Marital status | | |
| Unmarried | 22 (3.2) | 11 (1.5) |
| Married | 491 (71.3) | 579 (81.4) |
| Widowed | 174 (25.2) | 114 (16.1) |
| Divorced | 2 (0.3) | 7 (1.0) |
| Religion | | |
| Hindu | 537 (77.9) | 565 (79.5) |
| Muslim | 96 (13.9) | 97 (13.6) |
| Christian | 37 (5.4) | 34 (4.8) |
| Other | 19 (2.8) | 15 (2.1) |

non-vegetarian diet and literacy status were not statistically significantly related to risk of developing breast cancer in our study group.

A history of abortion and stillbirth among eligible cases and controls was also studied for the risk of breast cancer. Seventy-one cases and 97 controls reported one or more abortions. The relative risk for women with a history of abortion was 0.8 (CI 0.59–1.09) compared with those with no history of abortion, and this was not statistically significant. Ten cases and 12 controls had a history of stillbirths. The relative risk was 0.9 (CI 0.61–1.37) and the difference was not statistically significant.

The relative risk estimates for factors such as age at menarche, age at marriage, age at first pregnancy and number of pregnancies are presented in Table IV. Owing to the small number of cases in some of the categories, it was not possible to adjust for age and place of residence. Hence relative risks were calculated for an unadjusted group only. Age at menarche after 15 years compared with 14 years and below did not show statistically significant differences for breast cancer risk. Women married after 30 years of age showed a 2.5 excess risk of breast cancer compared with women married before 15 years of age ($P<0.01$) For women with a first pregnancy after 30 years of age the relative risk was 5.4 compared to women with a first pregnancy before 15 years of age. Three or more pregnancies was associated with

**Table II** Reproductive factors among cases and controls

| | *Cases* | *Controls* |
|---|---|---|
| Number | 689 | 711 |
| Average age at menarche[a] | 13.9 | 13.8 |
| Standard deviation | 1.3 | 1.4 |
| Average age at marriage[b] | 16.8 | 16.7 |
| Standard deviation | 4.8 | 4.2 |
| Average age at first pregnancy[c] | 20.4 | 19.8 |
| Standard deviation | 4.3 | 3.5 |
| Average number of pregnancies[d] | 4.3 | 4.5 |
| Standard deviation | 2.1 | 2.1 |

[a]In 13 controls and 15 cases, age at menarche was not recorded. [b]In six cases and four controls the age at marriage was unknown. [c]Eight cases and nine controls unknown. [d]In one case and one control the number of pregnancies was unknown.

**Table III** Relative risk (RR) estimate for factors and their confidence intervals

| *Risk factors studied* | *Cases factor/ non-factor* | *Controls factor/ non-factor* | *RR[a] (adjusted)* |
|---|---|---|---|
| Marital status | | | |
| Unmarried/ever married | 22/667 | 11/700 | 2.26[b] (1.01–5.06) |
| Parity status[c] | | | |
| Nulliparous/parous | 61/603 | 32/667 | 2.2*** (1.4–3.3) |
| Breast feeding[b] | | | |
| No/yes | 17/579 | 11/653 | 2.02 NS (0.8–4.9) |
| Stillbirth[d] | | | |
| Yes/no | 10/653 | 12/687 | 0.9 NS (0.6–1.4) |
| Abortion[e] | | | |
| Yes/no | 71/593 | 97/602 | 0.8 NS (0.6–1.1) |
| Food habits[f] | | | |
| Non-vegetarian/ vegetarian | 484/202 | 543/164 | 0.8 NS (0.6–2.1) |
| Literacy status Literate/illiterate | 295/394 | 326/385 | 1.1 NS (0.87–1.4) |

[a]Stratified for four age groups ($<35$, 35–44, 45–54 and $>55$ years) and three places of residence (Bombay, Maharashtra and other). [b]Seven cases and three controls not recorded. [c]Three cases and one control was not recorded. [d]Four cases and one control not recorded. [e]Three cases and one control not recorded. [f]Three cases and four controls not known. Figures in parentheses indicate lower and upper confidence interval. ***$P<0.001$. NS, not significant. Non-factor – reference category – RR = 1.0.

**Table IV** Relative risk (RR) estimate for factors and their confidence intervals

| *Risk factors studied* | *Cases* | *Controls* | *RR unadjusted* | *$\chi^2$ for trend* |
|---|---|---|---|---|
| Age at menarche (years) | | | | |
| ⩽14 | 277 | 293 | 1.0 | |
| 15 | 171 | 203 | 0.9 NS (0.7–1.2) | |
| 16 | 148 | 136 | 1.2 NS (0.9–1.5) | |
| 17 | 63 | 47 | 1.4 NS (0.9–2.1) | |
| 18 | 8 | 9 | 0.9 NS (0.3–2.5) | |
| 19 | 7 | 10 | 0.7 NS (0.3–1.9) | $P>0.05$ |
| Age at marriage (years) | | | | |
| ⩽14 | 194 | 188 | 1.0 | |
| 15–19 | 310 | 346 | 0.8 NS (0.7–1.1) | |
| 20–24 | 119 | 128 | 0.9 NS (0.7–1.2) | |
| 25–29 | 22 | 28 | 0.8 NS (0.4–1.4) | |
| 30 | 16 | 6 | 2.5** (1.0–6.6) | $P<0.05$ |
| Age at first pregnancy | | | | |
| ⩽14 | 17 | 28 | 1.0 | |
| 15–19 | 265 | 317 | 1.3 NS (0.7–2.6) | |
| 20–24 | 235 | 250 | 1.5 NS (0.8–2.9) | |
| 25–29 | 55 | 63 | 1.4 NS (0.7–2.9) | |
| 30 | 30 | 9 | 5.4** (2.2–13.9) | $P<0.004$ |
| No. of pregnancies | | | | |
| Nulliparous | 61 | 32 | 1.0 | |
| One | 45 | 33 | 0.7 NS (0.3–1.3) | |
| Two | 86 | 74 | 0.6 NS (0.4–1.0) | |
| Three | 115 | 121 | 0.5** (0.3–0.8) | |
| Four | 111 | 135 | 0.4** (0.3–0.7) | |
| Five | 94 | 119 | 0.4** (0.3–0.7) | |
| Six plus | 153 | 184 | 0.4** (0.3–0.7) | $P<0.0001$ |

NS, not significant. **$P<0.01$. Figures in parentheses indicate lower and upper confidence intervals.

a significant reduction in breast cancer risk compared with nulliparity.

### Discussion

Breast cancer is the second commonest cancer among females in developing countries, including India. Many case-control studies have been carried out in developed countries where breast cancer has been the most common cancer among females. It would be interesting to note whether established high-risk factors also play a significant role in a low-incidence area. An attempt has been made to identify high-risk groups and the role of reproductive factors in breast cancer.

Cases and controls were generally interviewed by our social investigators before medical examination. This helped to eliminate any interviewer bias in the collection of data.

Hospital controls were used instead of population controls. In the selection of controls, care was taken to include females without any history of either benign breast lesions or any gynaecological complaints. For a number of different reasons not all patients with breast cancer registered during the period could be interviewed. These are some of the limitations of the study which may or may not have affected the relative risk estimates.

The positive aspect is that the number of cases and controls is sufficient to detect a 2-fold increased risk level for factors with 90% power when such differences exist (Schlesselman, 1974).

Unmarried women and nulliparous women had a 2-fold increased risk for breast cancer. Also, late age at marriage (30 years and above) and late age at first pregnancy (30 years and above) were found to be risk factors for breast cancer. Multiparous women with three or more pregnancies had a 40-50% reduction in risk of breast cancer compared with nulliparous women. These findings are consistent with earlier reported studies from high-risk populations.

Paymaster and Gangadharan (1972) in a one-to-one matched case-control study on women from western India also showed that factors such as marital status, age at marriage, parity status, age at first delivery and number of pregnancies are associated with the risk of breast cancer.

The association of alcohol and dietary factors with breast cancer has also been reported in a high-incidence population (Schatzkin *et al.*, 1987; Willet *et al.*, 1987). In India, women in general do not indulge in alcohol in the same way as men. So, because of the negligible number of cases and controls with this habit, the effect of alcohol could not be studied. In our study we did not collect data on dietary factors, but information on type of food (i.e. vegetarian or non-vegetarian diet) consumed was collected for cases and controls. The risk level for non-vegetarians was lower than for vegetarians, but the difference was not statistically significant. Vegetarians who totally avoid animal meat, fish and poultry products generally consume less fat than non-vegetarians. In this context, the odds ratio was expected to be higher among the non-vegetarian group than among the vegetarian group since a diet with a high animal fat intake has been shown to increase the risk of breast cancer. Further studies are required to identify the association of dietary factors in breast cancer.

Moore *et al.* (1971) identified certain virus-like particles in the milk samples from the Parsis women in Bombay. However, further studies have not been done to confirm the viral aetiology (Gangadharan *et al.*, 1975).

The incidence of breast cancer is low in India compared with developed countries, but the rates are increasing (Yeole *et al.*, 1990). Cancer of the cervix uteri is the major leading site among females in most of the metropolitan registries in India, except in Greater Bombay, where for the last 10 years female breast cancer has been the leading site of cancer (Jussawalla *et al.*, 1992).

Recently Jayant (1986) reported that the increase in the incidence of breast cancer in Bombay is not due to a cohort effect, unlike the decrease in incidence of cervix cancer.

The trends in incidence rates for the Bombay population over the years 1964-85 show that crude, age-adjusted and truncated rates are increasing at the rate of 1-1.5% and in all the age groups except those aged 35-44 years (Yeole *et al.*, 1990). The increase in incidence of breast cancer can be partly explained by changes in lifestyle, such as an increase in the number of 'unmarried women', later age at marriage and consequent later age at first pregnancy. Further studies are necessary to explain the role of dietary factors in breast cancer. With the change in lifestyle, smaller families and better socioeconomic advancement, the incidence of breast cancer is bound to increase over the years. In a developing country like India, with a large female population in high-risk groups, known methods of early detection such as mass screening and compulsory mammography may not be economically viable. However, propagation of breast self-examination may be important in helping to combat this health problem.

The authors wish to thank the staff of the Division for their cooperation and assistance. Special thanks for the social investigators, Mrs Pushpa Peshotan and Mrs Rajani Vachharajani, who took great pains to interview cases and controls during the period of study. Our sincere thanks to Dr R.S. Rao, Director, Tata Memoral Hospital, for his constant encouragement and support.

### References

ADAMI, H.-O., ADAMS, G., BOYLE, P., EWERTZ, M., LEE, N.C., LUND, E., MILLER, A.B., OLSSON, H., STEEL, M., TRICHOPOULOS, D. & TULINIUS, H. (1990). Critical overviews of natural history, etiology, molecular biology and screening by mammography. Chapter II Breast Cancer Etiology. *Int. J. Cancer* (Suppl.) **5**, 22-39.

GANGADHARAN, P., JUSSAWALLA, D.J., RAO, D.N. & PAYMASTER, J.C. (1975). Multiple approaches (for cancer) to well defined population of Parsis. In *Proceedings of the XI International Cancer Congress, Florence*, Vol. 3, Bucalossi, P., Veronesi, U. & Cascinelli, N. (eds). Excerpta Medica: Amsterdam, pp. 18-25.

JAYANT, K. (1986). Cancers of the cervix, uteri and breast. Changes in incidence rates in Bombay over the last two decades. *WHO Bull.*, **64**, 431-435.

JUSSAWALLA, D.J., YEOLE, B.B. & NATEKAR, M.V. (1992). *Cancer Morbidity and Mortality in Greater Bombay - 1990*. Bombay Cancer Registry: Bombay.

KLEINBAUM, D.G., KUPPER, L.L. & MORGENSTERN, H. (1982). *Epidemiologic Research, Principles and Quantitative Methods*. Lifetime Learning publications: Belmont, CA.

MACMAHON, B., COLE, P., LIN, T.M., LOWE, C.R., MIRRA, A.P., RAVNIHAR, B., SALBER, E.J., VALAORAS, V.G. & YUASA, S. (1970*a*). Age at first birth and breast cancer risk. *WHO Bull.*, **43**, 209-221.

MACMAHON, B., LIN, T.M., LOWE, C.R., MIRRA, A.P., RAVNIHAR, B., SALBER, E.J., TRICHOPOULOS, D., VALAORAS, V.G. & YUASA, S. (1970*b*). Lactation and cancer of the breast. *WHO Bull.*, **42**, 185-194.

MANTEL, N. & HAENZEL, W. (1959). Statistical aspects of analysis of data from retrospective studies of disease. *J. Natl Cancer Inst.*, **22**, 719-748.

MOORE, D.H., CHARNEY, J., KARMARSKY, B., LASFARGUES, E.Y., SARKAR, N.H., BRENNAN, M.J., BURROWS, J.H., SIRSAT, S.M., PAYMASTER, J.C. & VAIDYA, A.B. (1971). Search for a human breast cancer virus. *Nature*, **229**, 611-614.

NCRP (NATIONAL CANCER REGISTRY PROGRAMME) (1992). *Biennial Report 1988-1989. An Epidemiological Study*. Indian Council of Medical Research: New Delhi.

PARKIN, D.M., PISANI, P. & FERLAY, J. (1993). Estimates of the worldwide incidence of eighteen major cancers in 1985. *Int. J. Cancer*, **54**, 594-606.

PAYMASTER, J.C. & GANGADHARAN, P. (1972). Some observations on the epidemiology of cancer of the breast in women in western India. *Int. J. Cancer*, **10**, 443-450.

SCHATZKIN, A., JONES, D.Y., HOOVER, R.N., TAYLOR, P.R., BRINTON, L.A., ZIEGLER, R.G., HARVEY, E.B., CARTER, C.L., LICITRA, L.M., DUFOR, M.C. & LARSON, D.B. (1987). Alcohol consumption and breast cancer in the epidemiologic follow-up study of the first national health and nutrition examination survey. *N. Engl. J. Med.*, **316**, 1169-1173.

SCHLESSELMAN, J.J. (1974). Sample size requirements in cohort and case-control studies of disease. *Am. J. Epidemiol.*, **99**, 381-384.

WILLETT, W.C., STAMPFER, M.J., COLDITZ, G.A., ROSNER, B.A., HENNEKENS, C.H. & SPEIZER, F.E. (1987). Moderate alcohol consumption and the risk of breast cancer. *N. Engl. J. Med.*, **316**, 1174-1180.

WYNDER, E.L., YASUYUKI, F., RANDALL, E.H., TAKESHI, H. & TOMOHIKO, H. (1991). Comparative epidemiology of cancer between the United states and Japan. A second look. *Cancer*, **67**, 746-749.

YEOLE, B.B., JAYANT, K. & JUSSAWALLA, D.J. (1990). Trends in breast cancer incidence in Greater Bombay: an epidemiological assessment. *WHO Bull.*, **68**, 245-249.